# The impact of OSCAR-IB quality control and manual segmentation on retinal atrophy rates in multiple sclerosis

**DOI:** 10.3389/fneur.2026.1817433

**Published:** 2026-08-12

**Authors:** Laura Stichaller, Nik Krajnc, Fabian Föttinger, Jan Philipp Nolte, Tobias Monschein, Markus Ponleitner, Barbara Kornek, Fritz Leutmezer, Stefan Macher, Paulus Rommer, Christiane Schmied, Karin Zebenholzer, Tobias Zrzavy, Gudrun Zulehner, Thomas Berger, Berthold Pemp, Gabriel Bsteh

**Affiliations:** 1 Department of Neurology, Medical University of Vienna, Vienna, Austria; 2 Comprehensive Center for Clinical Neurosciences and Mental Health, Medical University of Vienna, Vienna, Austria; 3 Department of Ophthalmology, Medical University of Vienna, Vienna, Austria

**Keywords:** GCIPL, multiple sclerosis, optical coherence tomography, OSCAR-IB, pRNFL, quality control

## Abstract

**Introduction:**

Inner retinal layer thinning, particularly of the peripapillary retinal nerve fiber layer (pRNFL) and the ganglion cell-inner plexiform layer (GCIPL), as measured by optical coherence tomography (OCT), is an established surrogate biomarker of neuroaxonal damage in multiple sclerosis; however, the influence of preprocessing strategies on longitudinal retinal change estimates remains unclear. We investigated the effects of OSCAR-IB quality control (QC) and manual segmentation correction on retinal layer change rates.

**Methods:**

People with MS (pwMS) with ≥ 2 OCT routine scans obtained ≥ 6 months apart were included. Annualized pRNFL and GCIPL thickness change was estimated using linear mixed-effects models across four preprocessing strategies: raw data, data after full OSCAR-IB QC, data after QC excluding the algorithm-criterion for segmentation, and data after manual segmentation correction. Longitudinal stability was evaluated using residual variance and random slope variance.

**Results:**

A total of 173 pwMS (mean age 34.6 years [8.5], 72.3% female) were included. Mean annualized pRNFL and GCIPL thickness change in raw data were −0.29%/year (0.29) and 0.14%/year (0.37), respectively. Compared with raw data, application of OSCAR-IB QC was associated with lower residual variance for both pRNFL (1.50 vs. 1.73) and GCIPL (0.65 vs. 0.78). Manual segmentation correction did not improve longitudinal stability for either retinal layer.

**Conclusion:**

OSCAR-IB QC was associated with improved longitudinal consistency of pRNFL and GCIPL change estimates in MS. In contrast, manual segmentation correction was not associated with additional benefit beyond exclusion-based QC, suggesting limited added value in routine clinical or large-scale research settings where feasibility and scalability are critical.

## 1 Introduction

Multiple sclerosis (MS) is characterized by the coexistence of focal inflammatory demyelinating events and a slowly evolving, diffuse process of neuroaxonal degeneration that may begin early in the disease course and, despite often remaining clinically silent initially, contributes substantially to irreversible disability and long-term outcomes (1, 2). Accordingly, sensitive biomarkers capable of capturing subtle, longitudinal neurodegenerative changes have become central to disease monitoring in people with MS (pwMS).

In this context, optical coherence tomography (OCT) has emerged as a non-invasive, high-resolution imaging modality that enables *in vivo* quantification of inner retinal layers thickness, providing a direct and biologically meaningful window into central nervous system neuroaxonal degeneration (3). Among OCT parameters, thinning of the peripapillary retinal nerve fiber layer (pRNFL) and the macular ganglion cell-inner plexiform layer (GCIPL) are well-established biomarkers associated with physical disability, cognitive impairment, and disease progression in MS (4–7). However, inner retinal layer thinning in MS is quantitatively small and often approaches the technical limits of OCT measurement precision, rendering it susceptible to variability introduced by acquisition-related artifacts, segmentation inaccuracies, and operator-dependent factors (8). These limitations are accentuated in longitudinal analyses, where even minor systematic errors may bias change estimates and compromise their clinical interpretability.

To mitigate such sources of variability, the OSCAR-IB quality control (QC) framework has been widely adopted to standardize OCT acquisition and improve measurement reliability (9–11). In practice, however, OSCAR-IB criteria are typically applied as a binary inclusion criterion, with scans classified as either passing or failing QC (4, 12–14). Although OSCAR-IB QC has been known to improve the test-rest reliability of cross-sectional retinal thickness measurements, its effect on longitudinal estimates of pRNFL and GCIPL change rates remains unknown (8, 15). This uncertainty is clinically relevant, as stringent exclusion-based approaches may substantially reduce data availability in routine practice, whereas *post hoc* manual correction of algorithm-related segmentation errors increases workload and introduces potential subjectivity.

Against this background, it remains unclear how exclusion-based versus correction-based preprocessing strategies influence longitudinal retinal change rates in MS. The aim of this study was therefore to systematically evaluate the impact of OSCAR-IB QC and manual segmentation correction on retinal change rates derived from routine OCT scans in pwMS. We hypothesized that OSCAR-IB QC would improve the longitudinal stability of retinal change estimates and that targeted manual segmentation correction might confer additional benefit beyond exclusion-based QC. By directly comparing these preprocessing approaches, we sought to provide evidence-based guidance for the optimized use of OCT in clinical routine and longitudinal MS research.

## 2 Methods

### 2.1 Patients

In this retrospective longitudinal study, patients from an ongoing prospective observational study conducted at the Department of Neurology and the Department of Ophthalmology and Optometry, Medical University of Vienna with relapsing MS according to 2017 McDonald criteria were included. Further inclusion criteria comprised: (i) age ≥ 18 years, (ii) availability of ≥ 2 OCT scans at least 6 months apart. Continuous adherence to a single DMT during follow-up was not required for inclusion. Eyes with diagnoses of ophthalmologic (i.e., high myopia [greater than −6 dpt], glaucoma, optic disc drusen, keratoconus, papilledema, epiretinal membrane, vitreomacular traction, or other vitreomacular interface abnormalities), neurologic, or drug-related retinal pathology unrelated to MS were excluded.

### 2.2 Optical coherence tomography

OCT imaging was performed by neuro-ophthalmologists using a single spectral-domain OCT device (Spectralis OCT, Heidelberg Engineering, Heidelberg, Germany; software Heidelberg eye explorer [HEYEX] version 6.16.6.0) in a dark room on both eyes without pupil dilatation. The investigators performing the OCT were blinded to MS clinical parameters. Due to the retrospective nature of the study, OCT examinations were acquired as part of routine clinical care and scan acquisition parameters were not completely standardized across all visits. For pRNFL measurement, a custom 12° (3.4 mm) ring scan centred on the optic nerve head was used (1,536 A-scans, automatic real-time tracking [ART]: 100 averaged frames) (16). The most frequently used protocol consisted of a 20° × 20° macular volume scan centred on the macula (512 A-scans, 25 B-scans, ART 16); however, additional protocols with different raster orientations, B-scan densities and ART settings were also included. GCIPL thickness was defined as the mean layer thickness of the four inner and outer quadrants of the circular grid centred around the foveola corresponding to the 3 mm and 6 mm rings as defined by the Early Treatment Diabetic Retinopathy Study, excluding the central 1-mm diameter zone from the volumetric scan (17). Image processing was conducted semi-automatically using the built-in proprietary HEYEX software (HRA viewing module version 6.16.6.0, Heidelberg Engineering).

QC of OCT scans was performed in accordance with the OSCAR-IB consensus criteria by a single trained physician (LS) following standardized instruction by an experienced rater (NK) (9). Each scan was systematically evaluated for all OSCAR-IB criteria using a rule-based approach, and scans were termed as either fulfilling (SFO) or not fulfilling OSCAR-IB criteria (SNFO) (8). Scans with algorithm-related segmentation errors (A-criterion) otherwise fulfilling OSCAR-IB criteria were identified separately to enable direct comparison of exclusion- and segmentation-based preprocessing strategies. Manual segmentation correction was performed blinded to clinical data and temporal scan order. In ring scans, the pRNFL boundaries were corrected (the inner limiting membrane [ILM] and the RNFL/GCL boundary); in macular scans, the boundaries of the ganglion cell layer (GCL) and inner plexiform layer (IPL) were corrected (the RNFL/GCL, GCL/IPL, and IPL/inner nuclear layer [INL] boundaries). All B-scans within each macular volume were systematically reviewed, and corrections were applied selectively to all with evident segmentation errors by a single trained grader (LS). Retinal layer thickness values measurements were extracted for four predefined preprocessing strategies: (i) raw OCT data without QC, (ii) data after exclusion of all scans not fulfilling the OSCAR-IB criteria (SNFO), (iii) data after exclusion of scans not fulfilling the OSCAR-IB criteria except for those failing solely the A-criterion (SNFO-A), and (iv) data after exclusion of scans not fulfilling the OSCAR-IB criteria except for those failing solely the A-criterion with additional manual correction (SNFO-A-corr).

Eyes with a history of unilateral ON within 6 months before baseline were excluded. Eyes with prior ON ≥ 6 months before baseline or evidence of subclinical ON, defined by inter-eye difference ≥5 μm or ≥5% for pRNFL and/or ≥4 μm or ≥4% for GCIPL, were eligible for inclusion (18, 19). Eyes developing clinical or subclinical ON during follow-up were excluded from longitudinal analyses.

### 2.3 Standard protocol approvals, patient consents and reporting

The study was approved by the ethics committee of the Medical University Vienna (ethical approval number: 1378/2020). Written informed consent was obtained from all study participants. This study adheres to the reporting guidelines outlined within the ‘Strengthening the Reporting of Observational Studies in Epidemiology’ (STROBE) Statement. The quantitative OCT study results were reported using the revised Advised Protocol for OCT Study Terminology and Elements (APOSTEL 2.0) recommendations (20).

### 2.4 Statistics

Statistical analysis was performed using R (version 4.5.2). Categorical variables were expressed in absolute frequencies and percentages, continuous variables were reported depending on their distribution as mean and standard deviation (SD) or as median with interquartile range (IQR). Continuous variables were tested for normal distribution using the Shapiro–Wilk normality test.

Annualized pRNFL and GCIPL thickness change was calculated using linear mixed-effects models fitted at the eye level using restricted maximum likelihood (REML), with time modelled continuously in years from baseline as the fixed effect (*β*, expressed in μm/year) and a random intercept and random slope for time per eye. Eyes were treated as independent units rather than nested within patients, as the proportion of patients contributing both eyes varied across preprocessing strategies. A nested sensitivity specification produced singular fits in five of eight datasets; results of the nested specification are provided in Supplementary Table 1. For descriptive analyses, pRNFL and GCIPL thicknesses were averaged across both eyes, excluding eyes with a history of ON. Percentage annualized change rates were calculated by dividing annualized pRNFL and GCIPL thickness change by baseline retinal layer thickness and multiplying by 100.

Longitudinal stability was assessed primarily by residual variance, with lower values indicating greater stability. Random slope variance was evaluated as a complementary measure of between-eye heterogeneity in change trajectories, with higher values reflecting greater differentiation of individual slopes. Comparisons were performed across the four preprocessing strategies: (i) raw data, (ii) SNFO, (iii) SNFO-A, and (iv) SNFO-A-corr.

Sensitivity analyses were conducted by (i) sequentially excluding scans failing individual OSCAR-IB criteria, and (ii) performing a within-eye analysis restricted to eyes contributing to the quality-restricted dataset. The significance level was set at a two-sided *p*-value < 0.05.

## 3 Results

**Figure 1:**
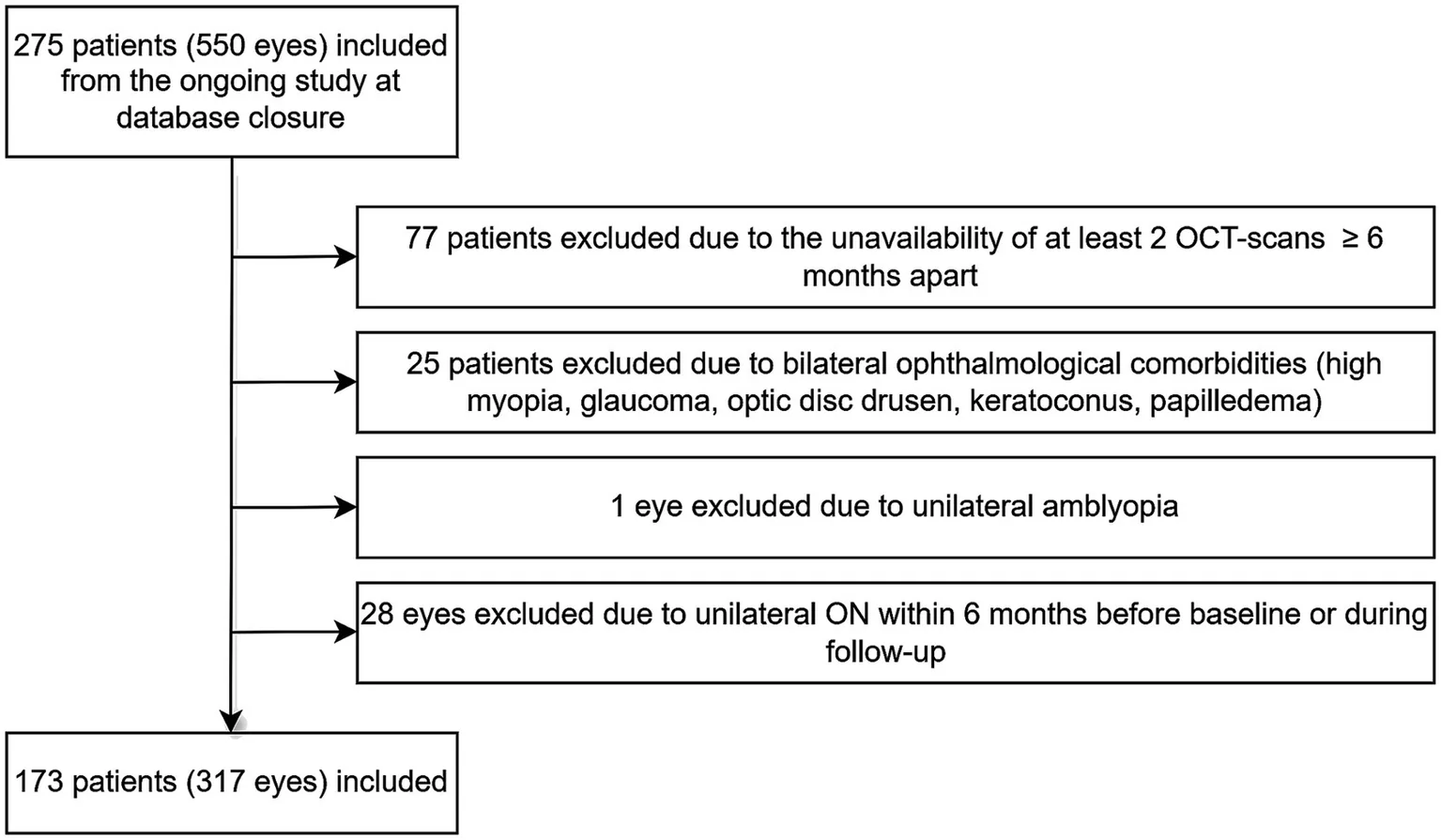
Flow chart of participant enrolment, exclusions, and final study cohort. OCT, optical coherence tomography. ON, optic neuritis.

Of the 275 patients enrolled in the ongoing study, 173 pwMS met the inclusion criteria for the primary analysis (mean age 34.6 years [8.5], 72.3% female). The detailed inclusion and exclusion process is shown in Figure 1. Demographic and clinical characteristics of the study cohort are summarized in Table 1.

**Table 1:** Demographics and clinical characteristics of the study cohort.

	All (*n* = 173)
Female^1^	125 (72.3%)
Age (years)^2^	34.6 (8.5)
Disease duration (months)^3^	31.0 (6.6–85.4)
EDSS^3^	1.0 (0.0–2.0)
ME-DMT^1^	46 (26.6%)
HE-DMT^1^	127 (73.4%)
pRNFL thickness (μm)^2^	96.4 (10.0)
GCIPL thickness (μm)^2^	69.2 (6.1)
Number of OCT scans (per patient) ^3^	4.0 (4.0–6.0)
OCT follow-up (months)^3^	20.9 (9.9–37.0)

EDSS, Expanded Disability Status Scale; GCIPL, ganglion cell-inner plexiform layer; HE-DMT, high-efficacy disease-modifying treatment (natalizumab, antiCD20 monoclonal antibodies, sphingosine-1-phospate-receptor modulators, cladribine). ME-DMT, moderate-efficacy disease-modifying treatment (dimethyl fumarate, teriflunomide, glatiramer acetate, interferon beta preparations); pRNFL, peripapillary retinal nerve fibre layer; pwMS, People with multiple sclerosis.

^1^Number (percentage), ^2^Mean (standard deviation), ^3^Median (interquartile range).

Of the 909 OCT routine ring scans analysed, 405 (44.6%) fulfilled the OSCAR-IB criteria (SFO). Of the remaining 504 (55.4%) scans (SNFO), 344 (37.8%) failed solely the A-criterion while fulfilling all other OSCAR-IB criteria and were therefore categorized as SNFO-A. Representative examples of A-criterion errors of varying severity are provided in Supplementary Figure 1.

**Figure 2:**
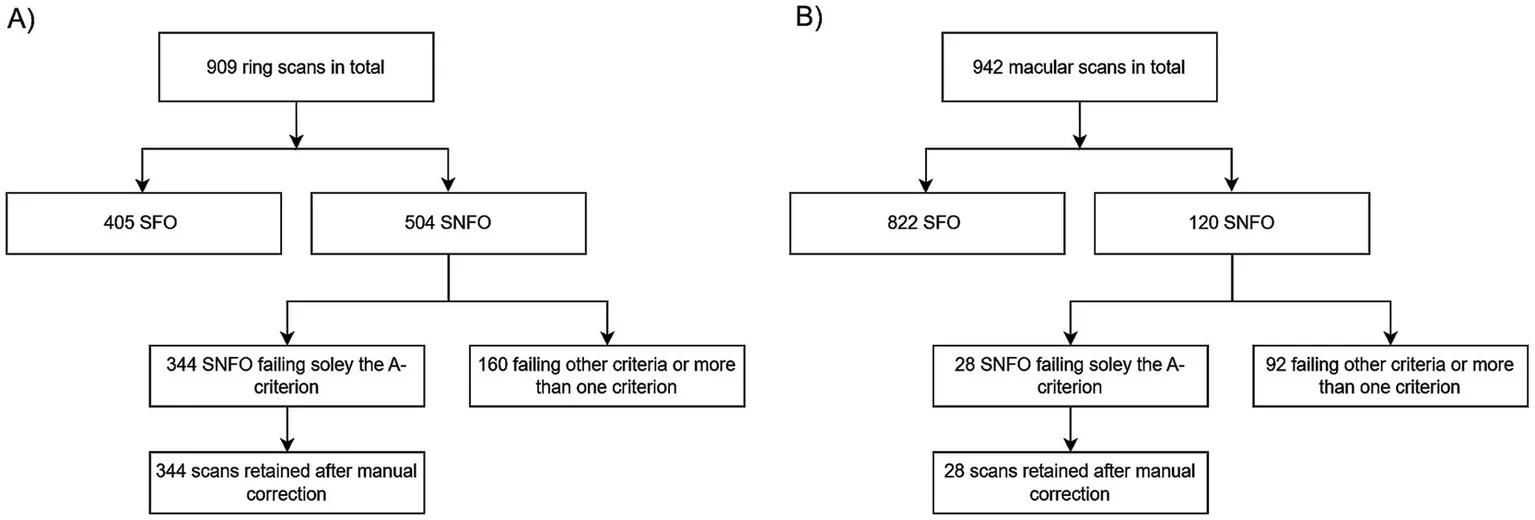
Allocation of ring (panel **A**) and macula scans (panel **B**) scans accross the four preprocessing strategies. Shown are the total number of scans, the numbers fulfilling OSCAR-IB criteria (SFO), scans not fulfilling the OSCAR-IB criteria (SNFO), scans failing only the A-criterion (SNFO-A), and scans retained after manual segmentation correction (SNFO-A-corr), together with the corresponding number of patients, eyes, and scans included in each analysis dataset.

**Figure 3:**
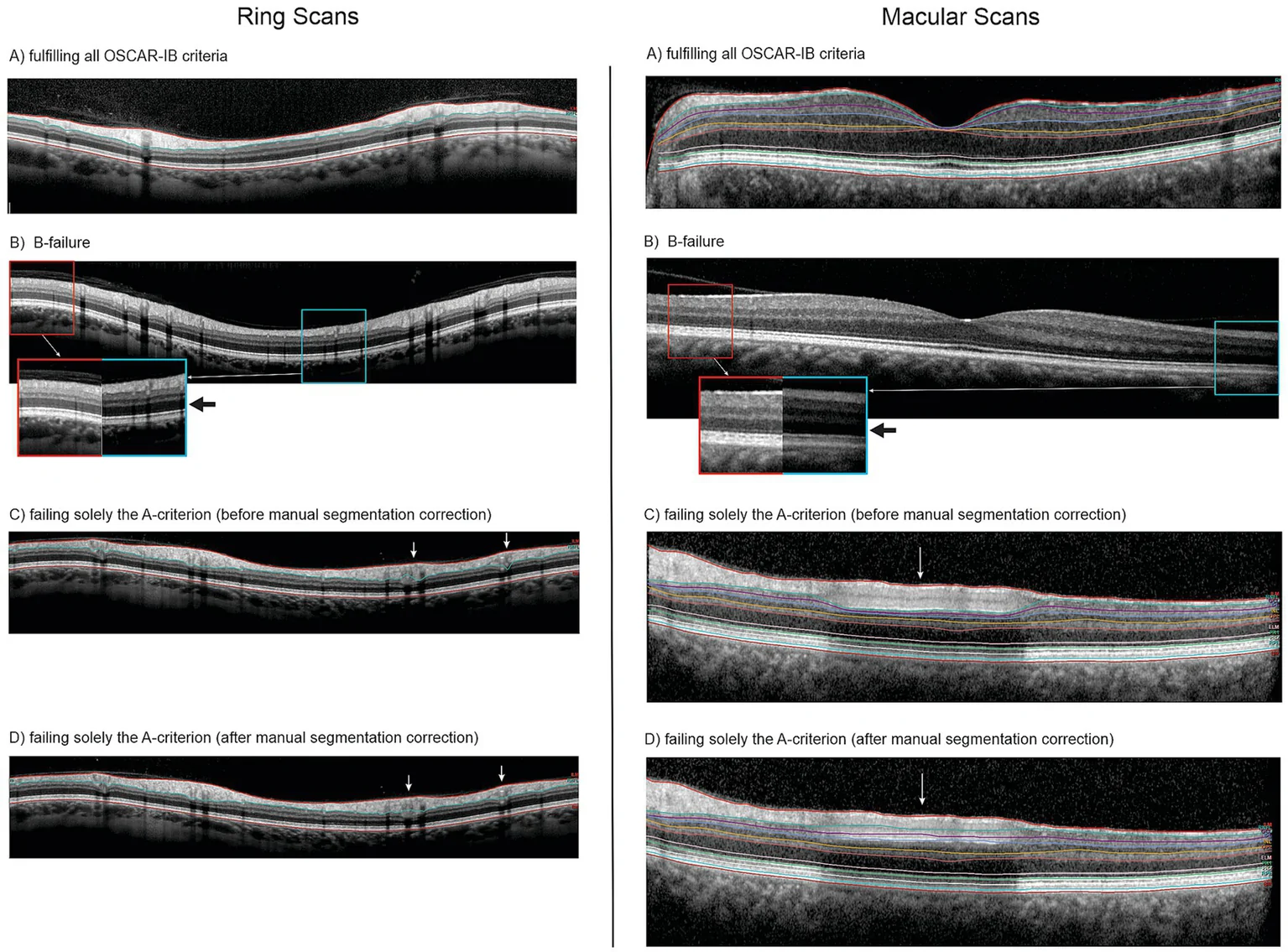
Representative ring (left) and macula (right) scans illustrating **(A)** a scan fulfilling the OSCAR-IB criteria, **(B)** a scan failing only the B-criterion (beam placement), **(C)** a scan failing solely the A-criterion (algorithm-related segmentation error), and **(D)** the same scan after manual segmentation correction.

In total, 942 OCT routine macula scans were evaluated. Of these, 822 scans (87.3%) fulfilled the OSCAR-IB criteria (SFO), while 120 scans (12.7%) did not (SNFO). Within this group, 28 scans (3.0%) failed solely the A-criterion but complied with all other OSCAR-IB criteria and were accordingly designated as SNFO-A. The distribution of OSCAR-IB QC failures for ring and macula scans is shown in Table 2. The allocation of scans across the four preprocessing strategies, shown separately for ring and macular scans, is summarized in Figure 2. Representative ring and macular scans illustrating the OSCAR-IB categories and an example of manual segmentation correction are shown in Figure 3.

**Table 2:** Distribution of OSCAR-IB quality control failures in ring and macular scans.

	Ring scan (*n* = 909)	Macula scan (*n* = 942)
O^1^	0 (0.0)	3 (0.3)
S^1^	7 (0.8)	27 (2.9)
C^1^	0 (0.0)	0 (0.0)
A^1^	417 (45.9)	46 (4.9)
R^1^	7 (0.8)	22 (2.3)
I^1^	3 (0.3)	13 (1.4)
B^1^	147 (16.2)	31 (3.3)
OSCAR-IB failed^1^	504 (55.4)	120 (12.7)
OSCAR-IB fulfilled^1^	405 (44.6)	822 (87.3)
1 failed criterion^1^	428 (47.1)	99 (10.5)
2 failed criteria^1^	75 (8.3)	20 (2.1)
3 failed criteria^1^	1 (0.1)	1 (0.1)
Only A failed	344 (37.8)	28 (3.0)
Only B failed	74 (8.1)	28 (3.0)

A, algorithm failure; B, beam placement; C, centration of scan; I, illumination; O, obvious problems; R, retinal pathology other than MS related; S, poor signal strength.

^1^Number (percentage).

Mean annualized pRNFL and GCIPL thickness change in raw data were −0.29%/year (0.29) and 0.14%/year (0.37), respectively. After full OSCAR-IB QC (SNFO), mean annualized pRNFL and GCIPL thickness change were −0.28%/year (0.36) and 0.18%/year (0.41), respectively. In SNFO-A scans, mean annualized pRNFL and GCIPL thickness change were −0.28%/year (0.40) and 0.17%/year (0.40), respectively, while after manual correction (SNFO-A-corr), mean annualized pRNFL and GCIPL thickness change were −0.21%/year (1.15) and 0.16%/year (0.41), respectively.

For annualized pRNFL thickness change, exclusion of all SNFO was associated with lower residual variance (1.50 vs. 1.73) and higher random slope variance (0.45 vs. 0.33) relative to the raw dataset, whereas SE increased slightly (0.10 vs. 0.06). For annualized GCIPL thickness change, exclusion of SNFO similarly reduced residual variance (0.65 vs. 0.78), with comparable random slope variance (0.22 vs. 0.20).

With respect to SNFO-A for annualized pRNFL thickness change, residual variance was lower than in the raw dataset (1.56 vs. 1.73) and random slope variance was higher (0.51 vs. 0.33), comparable to full SNFO exclusion (1.50; 0.45). For SNFO-A-corr, residual variance was similar to SNFO-A (1.57 vs. 1.56), while random slope variance was higher (0.71 vs. 0.51). SE remained low across approaches (0.06–0.08). For annualized GCIPL thickness change, all three QC strategies yielded comparable residual variances (SNFO 0.65, SNFO-A 0.67, SNFO-A-corr 0.66), all lower than the raw dataset (0.78), with random slope variances ranging from 0.20 to 0.22. SE remained stable across strategies (0.04–0.05). Detailed longitudinal stability metrics across preprocessing strategies are provided in Table 3.

**Table 3:** Longitudinal stability across preprocessing strategies for annualized pRNFL and GCIPL thickness change.

	Dataset	Patients (n)	Eyes (n)	Scans (n)	Median follow-up (months)	AIC	Beta (95% CI)	SE Beta	RV	RSV
Annualized pRNFL thickness change	Raw	172	315	909	20.8 (9.9–36.9)	4928.86	−0.28 (−0.39 to −0.16)	0.06	1.73	0.33
	Exclusion of SNFO	100	133	363	21.2 (10.5–37.2)	1994.06	−0.28 (−0.47 to −0.09)	0.10	1.50	0.45
	Exclusion of SNFO-A	155	260	701	19.4 (9.9–33.8)	3873.09	−0.28 (−0.43 to −0.14)	0.08	1.56	0.51
	Exclusion of SNFO-A-corr	155	260	701	19.4 (9.9–33.8)	3901.51	−0.31 (−0.47 to −0.15)	0.08	1.57	0.71
Annualized GCIPL thickness change	Raw	171	313	942	20.8 (9.9–36.5)	4244.45	0.10 (0.01 to 0.18)	0.04	0.78	0.20
	Exclusion of SNFO	161	273	785	20.8 (9.9–36.0)	3522.19	0.13 (0.04 to 0.22)	0.05	0.65	0.22
	Exclusion of SNFO-A	165	283	824	20.8 (9.8–36.1)	3685.58	0.12 (0.03 to 0.21)	0.05	0.67	0.21
	Exclusion of SNFO-A-corr	165	283	824	20.8 (9.8–36.1)	3681.82	0.12 (0.03 to 0.21)	0.05	0.66	0.22

AIC, akaike information criterion; CI, confidence interval; GCIPL, ganglion cell and inner plexiform layer; IQR, interquartile range; pRNFL, peripapillary retinal nerve fiber layer; RSV, random slope variance; RV, residual variance; SE, standard error; SNFO, scans not fulfilling the OSCAR-IB criteria; SNFO-A, scans not fulfilling the OSCAR-IB criteria except for those failing solely the A-criterion; SNFO-A-corr, scans not fulfilling OSCAR-IB criteria except for those failing solely the A-criterion with additional manual correction.

A sensitivity analysis excluding scans failing individual OSCAR-IB criteria and performing a within-eye analysis restricted to eyes contributing to the quality-restricted dataset did not significantly alter the overall results or the impact of individual variables (Supplementary Tables 2, 3).

## 4 Discussion

This study systematically evaluated the impact of OSCAR-IB QC and manual segmentation correction on retinal change rates in pwMS. Application of OSCAR-IB QC was associated with improved longitudinal consistency of both annualized pRNFL and GCIPL thickness change, with comparable reductions in residual variance across both retinal layers. In contrast, *post hoc* manual segmentation correction was not associated with additional benefit beyond exclusion-based QC. Together, these findings indicate that preprocessing strategies influence the reliability and interpretability of OCT-derived change rates in longitudinal MS studies.

Inner retinal layer thinning has been increasingly recognized as a marker of diffuse, subclinical neuroaxonal loss in MS, being associated with brain atrophy, disability accumulation, and PIRA (4–7). Detecting such subtle, biologically meaningful changes requires the ability to distinguish true longitudinal atrophy from technical variability. Early OCT studies and subsequent consensus efforts therefore emphasized the importance of rigorous, standardized QC to ensure that observed retinal thinning reflects neuroaxonal loss rather than acquisition artefacts or segmentation errors. Within this framework, the OSCAR-IB criteria were developed to harmonize scan quality assessment across centres and devices, thereby improving reproducibility and interpretability of OCT measures in MS research (8–10).

For GCIPL, exclusion of scans failing OSCAR-IB was associated with a reduction in residual variance, indicating less measurement variability. Residual variance was comparable across the three QC strategies, suggesting that for GCIPL the inclusion or exclusion of scans with algorithm-related segmentation errors has only a negligible effect on longitudinal stability. GCIPL thickness is derived from macular volume scans and represents an aggregate measure across multiple spatial regions (21). While such spatial averaging may mitigate the influence of isolated, localized acquisition artifacts, it does not eliminate susceptibility to broader scan-quality issues, including signal attenuation, motion artifacts, or systematic segmentation inaccuracies. From a methodological perspective, these findings suggest that GCIPL measurements remain sensitive to scan quality, supporting the use of rigorous and standardized QC procedures, particularly in multicentre and real-world settings characterized by increased heterogeneity and technical variability.

For pRNFL, the effect of preprocessing followed a similar pattern. Exclusion of scans failing OSCAR-IB criteria was associated with a comparable reduction in variability. pRNFL thickness is derived from a single peripapillary ring scan, rendering it particularly susceptible to scan decentration, minor deviations in ring placement, signal attenuation, and motion artifacts. This intrinsic vulnerability is reflected in the distribution of QC failures in our dataset, where ring scans exhibited a substantially higher overall OSCAR-IB failure rate than macular scans (55.4% vs. 12.7%), largely driven by algorithm-related segmentation errors. This high rate partly reflects our stringent application of the A-criterion, under which local segmentation displacements caused by peripapillary vessel shadows were also classified as failures when they measurably affected layer thickness. The high prevalence of segmentation failures in ring scans further supports the sensitivity of peripapillary measurements to acquisition and boundary-detection artefacts, providing a structural explanation for the differential impact of preprocessing strategies observed in our study.

Notably, for pRNFL, exclusion of low-quality scans was accompanied by increased random slope variance, suggesting improved discrimination of individual slope patterns. This likely reflects reduced measurement noise, allowing underlying between-eye differences to emerge more clearly. No comparable pattern was observed for GCIPL, likely owing to the smaller magnitude of change detectable over the observation period. Manual segmentation correction had little effect on residual variance for either retinal layer. Consistent with previous studies that have reported comparable reproducibility of automated and expert manual segmentation in cross-sectional OCT analyses (22, 23), our findings suggest that selective manual correction offers limited added value in longitudinal analyses. Given its resource-intensive nature, manual segmentation is unlikely to provide sufficient benefit to justify its use in routine clinical practice or large-scale multicenter studies.

Several limitations merit consideration. The single-centre design within a highly specialized setting using a single OCT platform (Heidelberg Engineering®) with a largely standardized protocol reduces technical variability and measurement error. Such sources of variability may be more substantial when different OCT devices or acquisition protocols are used, which may limit generalizability of our findings. As automated segmentation performance and failure patterns differ across OCT platforms, both the observed failure rates and the limited added value of manual correction may not translate directly to other devices. This also applies within our cohort, where macular acquisition parameters varied between visits in a proportion of patients. Moreover, the study population is predominantly composed of young individuals, largely of Caucasian background, with relatively short disease duration and active MS, which restricts the applicability of the results to other ethnicities, older patients, those with longer-standing disease, and more progressive disease courses. Older patients and those with more advanced disease may present greater segmentation challenges, potentially affecting both quality-control failure rates and the impact of the preprocessing strategies evaluated. Importantly, potentially confounding comorbidities were systematically excluded, including severe myopia, optic disc drusen, and ophthalmological, neurological, systemic, or medication-related causes of retinal damage unrelated to MS. Consequently, the generalizability of the results may be limited for populations not meeting these criteria. For GCIPL, the estimated annualized change was positive rather than negative, indicating apparent thickening. As GCIPL thickening is not biologically expected, this finding is unlikely to represent true retinal change. Given the short follow-up and the predominance of HE-DMT in our cohort, the expected rate of GCIPL thinning is relatively small. Consistent with this, the observed change of approximately 0.1 μm/year was below the reported test–retest variability of Spectralis GCIPL measurements (15). Besides, macular acquisition parameters were not fully standardized across follow-up, with changes in B-scan density, ART averaging, and raster orientation occurring in a subset of eyes (24). Furthermore, OSCAR-IB quality control classification and manual segmentation correction were performed by a single grader, and rater reliability was not formally assessed. Although all procedures followed standardized protocols under expert supervision, this should be considered when interpreting the findings. Nevertheless, the use of a well-characterized cohort, standardized acquisition on a single device, rule-based QC procedures, and within-eye sensitivity analyses ensured high internal validity and enabled a rigorous comparison of preprocessing strategies.

In summary, OSCAR-IB QC was associated with improved longitudinal consistency of pRNFL and GCIPL change estimates in MS, whereas manual segmentation correction was not associated with additional benefit. These findings support the use of standardized, exclusion-based quality control strategies in longitudinal OCT studies, contributing to reliable and reproducible retinal change quantification in MS.

## Data Availability

Data supporting the findings of this study are available from the corresponding author upon reasonable request by a qualified researcher and upon approval by the ethics committee of the qualified researcher’s institution and the data clearing unit of Medical University Vienna.
