# Supplementary material for: The impact of OSCAR-IB quality control and manual segmentation on retinal atrophy rates in multiple sclerosis
Source: Front Neurol. 2026 Aug 12;17:1817433. doi: 10.3389/fneur.2026.1817433 (PMC13506471; doi:10.3389/fneur.2026.1817433)
Supplement: Supplementary file 1 [file Supplementary_file_1.DOCX]

**Supplementary Table 1.** Nested specification of the linear mixed-effects model for the main analysis datasets, with random intercepts and slopes per patient and per eye nested within patient. AIC: Akaike Information Criterion, CI: confidence interval, GCIPL: ganglion cell and inner plexiform layer, IQR: interquartile range, pRNFL: peripapillary retinal nerve fiber layer, RSV eye: eye-within-patient random slope variance, RSV patient: patient-level random slope variance, RV: residual variance, SE: standard error, SNFO: scans not fulfilling the OSCAR-IB criteria, SNFO-A: scans not fulfilling the OSCAR-IB criteria except for those failing solely the A-criterion, SNFO-A-corr: scans not fulfilling OSCAR-IB criteria except for those failing solely the A-criterion with additional manual correction.

|  | Dataset | Patients (% single eye) | Eyes (n) | Scans (n) | Median follow-up (months) | Beta (95% CI) | SE Beta | AIC | RV | RSV eye | RSV patient | Fit status |
| --- | --- | --- | --- | --- | --- | --- | --- | --- | --- | --- | --- | --- |
| Annualized pRNFL thickness change | Raw | 172 (16.9%) | 315 | 909 | 20.8 (9.9-36.9) | -0.27 (-0.42 to -0.12) | 0.08 | 4794.43 | 1.61 | 0.004 | 0.439 | Singular |
|  | SNFO | 100 (67.0%) | 133 | 363 | 21.2 (10.5-37.2) | -0.24 (-0.46 to -0.02) | 0.11 | 1947.50 | 1.44 | 0.049 | 0.461 | Converged |
|  | SNFO-A | 155 (32.3%) | 260 | 701 | 19.4 (9.9-33.8) | -0.26 (-0.44 to -0.08) | 0.09 | 3776.70 | 1.50 | 0.047 | 0.562 | Converged |
|  | SNFO-A-corr | 155 (32.3%) | 260 | 701 | 19.4 (9.9-33.8) | -0.28 (-0.48 to -0.09) | 0.10 | 3804.35 | 1.52 | 0.126 | 0.660 | Converged |
| Annualized GCIPL thickness change | Raw | 171 (17.0%) | 313 | 942 | 20.8 (9.9-36.5) | 0.06 (-0.06 to 0.17) | 0.06 | 4104.67 | 0.71 | 0.001 | 0.292 | Singular |
|  | SNFO | 161 (30.4%) | 273 | 785 | 20.8 (9.9-36.0) | 0.09 (-0.02 to 0.21) | 0.06 | 3420.55 | 0.60 | 0.000 | 0.288 | Singular |
|  | SNFO-A | 165 (28.5%) | 283 | 824 | 20.8 (9.8-36.1) | 0.09 (-0.03 to 0.20) | 0.06 | 3574.33 | 0.61 | 0.000 | 0.292 | Singular |
|  | SNFO-A-corr | 165 (28.5%) | 283 | 824 | 20.8 (9.8-36.1) | 0.08 (-0.03 to 0.20) | 0.06 | 3566.90 | 0.60 | 0.000 | 0.303 | Singular |

**Supplementary Table 2.** Sensitivity analysis retaining scans that failed only the OSCAR-IB beam placement criterion (B). AIC: Akaike Information Criterion, CI: confidence interval, GCIPL: ganglion cell and inner plexiform layer, IQR: interquartile range, pRNFL: peripapillary retinal nerve fiber layer, RSV: random slope variance, RV: residual variance, SE: standard error, SNFO-B: scans not fulfilling the OSCAR-IB criteria except for those failing solely the B-criterion, SNFO-A-corr-B: scans not fulfilling OSCAR-IB criteria except for those failing solely the B-criterion with additional manual correction.

|  | Dataset | Patients (n) | Eyes (n) | Scans (n) | Median follow-up (months) | Beta (95% CI) | SE Beta | AIC | RV | RSV |
| --- | --- | --- | --- | --- | --- | --- | --- | --- | --- | --- |
| Annualized pRNFL thickness change | Raw | 172 | 315 | 909 | 20.8 (9.9-36.9) | -0.28 (-0.39 to -0.16) | 0.06 | 4928.86 | 1.73 | 0.33 |
|  | SNFO-B | 107 | 144 | 417 | 21.5 (10.1-37.3) | -0.25 (-0.41 to -0.09) | 0.08 | 2209.31 | 1.45 | 0.29 |
|  | SNFO-A-corr-B | 155 | 272 | 759 | 18.6 (9.8-34.5) | -0.29 (-0.43 to -0.14) | 0.07 | 4150.75 | 1.56 | 0.58 |
| Annualized GCIPL thickness change | Raw | 171 | 313 | 942 | 20.8 (9.9-36.5) | 0.10 (0.01 to 0.18) | 0.04 | 4244.45 | 0.78 | 0.20 |
|  | SNFO-B | 165 | 282 | 822 | 20.4 (9.9-36.8) | 0.11 (0.02 to 0.20) | 0.05 | 3666.90 | 0.63 | 0.24 |
|  | SNFO-A-corr-B | 168 | 292 | 861 | 20.4 (9.9-36.8) | 0.10 (0.01 to 0.19) | 0.05 | 3826.39 | 0.65 | 0.24 |

**Supplementary Table 3.** Sensitivity analysis restricted to eyes contributing to the quality-controlled dataset. AIC: Akaike Information Criterion, CI: confidence interval, GCIPL: ganglion cell and inner plexiform layer, IQR: interquartile range, pRNFL: peripapillary retinal nerve fiber layer, RSV: random slope variance, RV: residual variance, SE: standard error, SNFO-A-matched: scans not fulfilling the OSCAR-IB criteria except for those failing solely the A-criterion, SNFO-A-corr-matched: scans not fulfilling OSCAR-IB criteria except for those failing solely the A-criterion with additional manual correction.

|  | Dataset | Patients (n) | Eyes (n) | Scans (n) | Median follow-up (months) | Beta (95% CI) | SE Beta | AIC | RV | RSV |
| --- | --- | --- | --- | --- | --- | --- | --- | --- | --- | --- |
| Annualized pRNFL thickness change | Raw-matched | 100 | 133 | 416 | 22.8 (12.0-40.9) | -0.26 (-0.43 to -0.08) | 0.09 | 2212.23 | 1.69 | 0.36 |
|  | SNFO | 100 | 133 | 363 | 21.2 (10.5-37.2) | -0.28 (-0.47 to -0.09) | 0.10 | 1994.06 | 1.50 | 0.45 |
|  | SNFO-A-matched | 100 | 133 | 370 | 22.1 (11.3-37.6) | -0.25 (-0.44 to -0.06) | 0.10 | 2026.22 | 1.50 | 0.49 |
|  | SNFO-A-corr-matched | 100 | 133 | 370 | 22.1 (11.3-37.6) | -0.28 (-0.47 to -0.09) | 0.10 | 2022.78 | 1.51 | 0.45 |
| Annualized GCIPL thickness change | Raw-matched | 161 | 273 | 848 | 21.6 (9.9-37.2) | 0.12 (0.03 to 0.21) | 0.05 | 3786.80 | 0.77 | 0.21 |
|  | SNFO | 161 | 273 | 785 | 20.8 (9.9-36.0) | 0.13 (0.04 to 0.22) | 0.05 | 3522.19 | 0.65 | 0.22 |
|  | SNFO-A-matched | 161 | 273 | 801 | 21.0 (9.9-36.3) | 0.12 (0.03 to 0.21) | 0.05 | 3577.20 | 0.66 | 0.21 |
|  | SNFO-A-corr-matched | 161 | 273 | 801 | 21.0 (9.9-36.3) | 0.11 (0.02 to 0.20) | 0.05 | 3577.27 | 0.66 | 0.22 |

**Supplementary Figure 1.** Representative ring (left) and macula (right) scans illustrating A-criterion segmentation errors of varying severity, shown before (top) and after (bottom) manual segmentation correction. For ring scans, (A) and (B) show representative mild-to-moderate A-failures, and (C) a severe A-failure. For macular scans, (A) shows a mild, (B) a moderate, and (C) a severe A-failure. White arrows indicate the location of segmentation deviations. For pRNFL ring scans, mild and moderate A-failures fell along a continuum without consistent distinction into discrete severity categories; two examples are shown to illustrate this spectrum.
